# Is There a Key Primer for Amplification of Core Land Plant DNA Barcode Regions ( *rbcL* and *matK* )?

**DOI:** 10.1002/ece3.70961

**Published:** 2025-02-16

**Authors:** Leonardo C. J. Corvalán, Amanda A. de Melo‐Ximenes, Larissa R. Carvalho, Carlos de M. e Silva‐Neto, José A. F. Diniz‐Filho, Mariana P. de C. Telles, Rhewter Nunes

**Affiliations:** ^1^ Laboratório de Genética Biodiversidade—Universidade Federal de Goiás Goiânia Goiás Brazil; ^2^ Laboratório de Bioinformática e Biodiversidade, Instituto Acadêmico de Ciências da Saúde e Biológicas Universidade Estadual de Goiás—Campus Oeste—UnU de Iporá Iporá Goiás Brazil; ^3^ Instituto Federal de Goiás Polo de Inovação Goiânia Goiás Brazil; ^4^ Departamento de Ecologia ICB, Ecologia UFG, ICB, UFG Goiânia Goiás Brazil; ^5^ Escola de Ciências Médicas e da Vida Pontifícia Universidade Católica de Goiás Goiânia Goiás Brazil

**Keywords:** annealing evaluation, DNA metabarcode, *in silico* PCR, molecular identification, primer coverage

## Abstract

The DNA barcode is a technique for molecular identification of species. Two core genes, *matK* and *rbcL*, are widely used for land plants. In this technique, the selection of primers is a fundamental step for the success of amplification. Then, we aim to evaluate the primer amplification capability for the DNA barcode regions *rbcL* and *matK*. We extracted primer sequences from DNA barcode studies in the Web of Science and used chloroplast genome sequences from NCBI for *in silico* PCR tests using OpenprimeR. Physicochemical properties of *in silico* PCR were evaluated using OpenprimeR. Our literature review resulted in 366 and 489 different *rbcL* and *matK* primers. These were tested in 8665 sequences, 8463 species from 98 orders. Evaluating only the primer and sequence match, the primers with the highest number of sequences covered were 96.39% and 93.81% forward and reverse for *rbcL*, and 91.56% and 61.62% forward and reverse for *matK*. No universal primer for all land plants was found, but two *rbcL* primer pairs could amplify > 99% of the sequences. In contrast to the results obtained for the *matK* region, the 10 pairs optimized for the greatest coverage of sequences were not covered by > 85% of the sequences. Therefore, it is advisable to pay attention when selecting primers for the *matK* region and the need to develop new primers. Here, we recommend a set of primers to cover the largest number of sequences and orders.

## Graphical abstract

We use *in silico* PCR to select and measure the amplification capability of primer. We tested 366 and 489 published primers for the rbcL and matK regions, respectively, using sequences from 8463 different land plant species. Our results indicate a set of primers that effectively cover all land plant sequences, particularly for the rbcL region. The rbcL region showed the potential for a universal primer, with two pairs of primers covering > 99% of sequences. In contrast, the amplification of the matK region proved challenging; even using > 10 pairs of primers, we covered < 85% of sequences. Thus, we could not identify a universal primer for math, and we recommend using primers targeting lower phylogenetic levels.

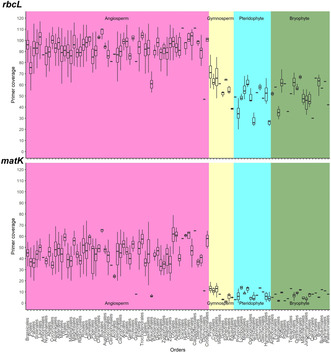

## 1 Introduction

DNA barcoding is a method used to identify species at the molecular level. It is based on extracting a DNA fragment from a sample, amplifying and sequencing it, and then comparing the resulting sequence to a database. This molecular identification approach has been used in plant science to understand phylogenetic relationships in the community, for phylogenetic reconstructions, to identify and delimit species, to understand species interactions, and in forensic applications (Kress 2017). For example, DNA barcode is used in forensic cases involving the illegal sale of timber and the verification of species that make up products such as teas, processed fruits, and oils (Apeti and N'Doua 2023; Kress 2017). Furthermore, the combination of molecular and anatomical technologies can be used to certify the origin of forest products, helping to reduce illegal activities (Deklerck 2023).

The DNA barcode was proposed in 2003, using a single region to distinguish species (Hebert et al. 2003a, 2003b). Initially, the main focus of the technology was on animal barcodes, which led to a consensus on the use of the gene cytochrome c oxidase subunit I (COI) (Hebert et al. 2004, 2003a, 2003b), a mitochondrial gene that constitutes the mitochondrial oxidative phosphorylation system fundamental to cellular respiration (Saraste 1990). In particular, the COI gene did not show the desired characteristics for a plant barcode due to lower plant mitochondrial genome (mtDNA) substitution rates compared to animal mtDNA (Kress et al. 2005). The molecular history and evolution of the plant genome, especially the organellar genome, did not accept the proposal of a single gene as a barcode (CBOL Plant Working Group 2009). However, the Consortium for the Barcode of Life (CBOL) Plant working group proposed two core barcode regions, in the chloroplast genome (cpDNA), and the *rbcL* and *matK* genes (CBOL Plant Working Group 2009).

The *rbcL* and *matK* genes are present in the cpDNA of most land plants. The exception is the parasitic plants, which had a reduced chloroplast genome size and a loss of genes associated with photosynthesis (Bungard 2004). This reduction in the photosynthesis genes is a result of the development of other ways of obtaining energy (Bungard 2004). The ribulose‐1,5‐bisphosphate carboxylase/oxygenase large subunit (*rbcL*) gene encodes the large subunit protein of ribulose bisphosphate carboxylase, a fundamental molecule for photosynthesis (Rhodes et al. 1980). The maturase K (*matK*) gene is involved in intron splicing (Neuhaus and Link 1987). Both genes play a critical role in the survival of autotrophic land plants (Neuhaus and Link 1987; Rhodes et al. 1980).

After 15 years of research on plant barcodes, there is still no consensus on a standard set of primers suitable for each group of land plants across different hierarchical levels. However, several universal primers have been developed that can amplify sequences for a wide range of species (Dunning and Savolainen 2010; Heckenhauer et al. 2016; Li et al. 2011). The development of primers depends on the database used and the characteristics of the regions (Yu et al. 2011). However, although the database has grown significantly over the past decades, the majority of available sequences still come from cultivated plants and temperate biodiversity (Wang et al. 2024). Another point is that the region used as a barcode must have the nucleotide difference between species and still be flanked by conserved regions used for primer annealing. Finding these conserved regions can be challenging in phylogenetically distant species, so species‐specific and order‐specific primers have been developed (Dunning and Savolainen 2010). Using more specific primers may fail to recover a broader taxonomic group in metabarcoding.

In this context, using the use of *in silico* PCR for primer selection can reduce the time and cost of primer testing (de Melo et al. 2021; Kreer et al. 2020). In addition, these approaches use templates (nucleotide sequences used as the base for testing the *in silico* annealing of primers) obtained from public databases, making it possible to compare data from groups with the most extensive distribution without the collected costs. Also, a large number of primers have been described as universal primers, and *in silico* tests to select the primers with the most extensive taxonomic coverage have not been performed and applied to differentiate taxonomic groups.

Because selecting a primer set is a critical aspect of barcoding and metabarcoding projects, an *in silico* test of the currently used primers was performed to address this issue. The investigation focused on two main questions: (1) Who are the universal primers? (2) How many primer pairs do we need to amplify the entire biodiversity of land plants? Answering such questions would be an important step toward mitigating important Linnean and Darwinian components of biodiversity shortfalls (Hortal et al. 2015). Thus, this paper aims to review the primers commonly used in the literature, to identify the primer with the largest coverage and the optimal primer set to amplify an extensive number of orders.

## 2 Materials and Methods

### 2.1 Primer Data

The primer sequences were obtained by a systematic search in the bibliography of scientific papers, using the following search algorithm: (ALL = (barcoding) OR ALL = (Metabarcoding) OR ALL = (Molecular identification) OR ALL = (eDNA) OR ALL = (e‐DNA)) AND ALL = (plant*) AND ((ALL = rbcL) OR ALL = (RuBisCO) OR ALL = (ribulose 1,5‐bisphosphate carboxylase) OR ALL = (matK) OR ALL = (maturases) OR ALL = (Maturase K)) in the Web of Science database. The search algorithm aimed to retrieve all articles using barcodes of the *rbcL* and *matK* genes. The database search was performed in May 2022, and only full research and review papers were evaluated. The primer sequences were manually extracted. If the primer sequence was not directly reported, it was located within the original article if cited. During manual inspection, papers were excluded if they lacked primer information, did not focus on land plants, were unavailable, or did not study the *matK* or *rbcL* genes.

### 2.2 rbcL and matK Sequences

To recover the complete gene sequence, we searched for complete chloroplast genomes in the RefSeq database. The RefSeq is a reference sequence database from the National Center for Biotechnology Information (NCBI). We used the keywords: (chloroplast[All Fields] AND complete genome[All Fields]) AND plastid[filter] AND (plants[filter] AND refseq[filter]) AND “Embryophyta”[Organism] AND chloroplast[filter]. We retrieved all land plant complete chloroplast sequences and annotations (GenBank file) available in RefSeq. We extracted the gene sequence using a Biopython package (Cock et al. 2009).

The sequence of *matK* and *rbcL* was polished according to the following pipeline: (1) The chloroplast genome must have the annotation of both genes, (2) the sequence of the genes must be 1000 bp or longer, and (3) the sequence must not have 5 or more consecutive Ns.

### 2.3 *In Silico* Primer Annealing Evaluation

Primer coverage analysis was conducted for each gene using the R package “OpenprimeR” (Kreer et al. 2020). *In silico* annealing, performance was evaluated separately for each gene. We used the default settings of the package, except for the primer size, which was defined between 18 and 30 bp, and the prohibition of mismatches on the last seven bases of their 3′ end. Due to the limited amount of data that could be loaded, we split the template data (sequences) into four parts for analysis. The OpenprimeR outputs were merged, and the figures were plotted in the R language (R Core Team 2000). The same package was used to evaluate the physicochemical properties of the primers.

To select the primers with the highest taxonomic coverage, we filtered the primers that did not meet the following criteria: (1) The primer must cover at least one sequence (primer_coverage, min = 1), (2) the GC clamp must have a maximum of 4 (GC_clamp, min = 0; max = 4), (3) the GC ratio must be between 30% and 70% (gc_ratio, min = 0.3; max = 0.7), (4) the maximum number of homopolymers accepted was 6 (no_runs, min = 0; max = 6), (5) the number of dinucleotide repeats must be < 6 (no_repeats, min = 0; max = 6), (6) the minimum free energy for self‐dimerization was −7 kcal/mol (self_dimerization, min = −7 kcal/mol), (7) the melting temperature was accepted in the range of 50°C–70°C (melting_temp_range, min = 50°C; max = 70°C), and (8) the minimum energy for the secondary structure was −1 kcal/mol (secondary_structure, min = −1 kcal/mol). The primers with the highest coverage were then selected.

### 2.4 Identifying the Optimal Set of Primers

The data used in the analyses consist of all primers that passed eight constraints to filter primers (described in *in silico* primer annealing assessment). We used a sequential approach for identifying the set of primers that covered the largest number of sequences and orders. We started by combining all possible combinations of two primers and identifying the number of sequences and orders covered. From this combination with two primers, we combine this set of two primers with individual primers for the identification of the number of sequences and orders covered. Therefore, the set of three primers with the largest number of sequences and orders covered were selected. This process was repeated until we obtained a set of 10 primers using an R script. In this process, we analyzed separately the forward and reverse primers, the covered sequences, and the covered orders.

## 3 Results

### 3.1 Primer Information

The search in the Web of Science found 1182 papers, of which 647 directly or indirectly reported primer sequences (Table S1). In total, 175 and 191 different forward and reverse primers were recovered for *rbcL* (Table S2). A large number of different primers were detected for the *matK* gene with 248 and 241 different primers, respectively (Table S2). The primer with the same sequence and different name or reference was adjusted to keep the oldest name or reference.

**FIGURE 1:**
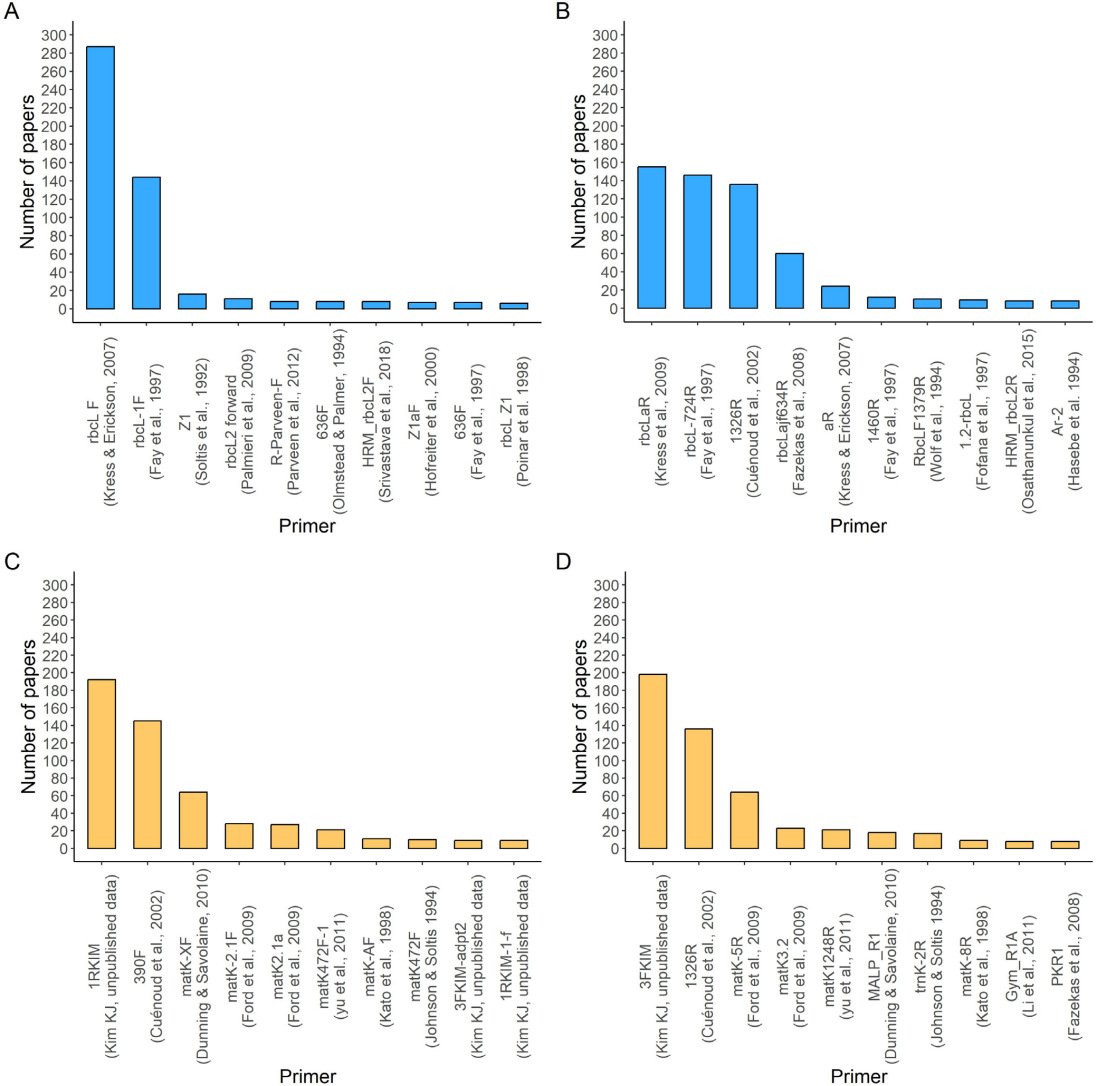
Top 10 *rbcL and matK* most used primers. (A) Forward *rbcL* primers. (B) Reverse *rbcL* primers. (C) Forward *matK* primers. (D) Reverse *matK* primers.

The 366 *rbcL* primers were derived from 121 different references. The most commonly used forward primers for *rbcL* were *rbcL F* (Kress and Erickson 2007), rbcL‐1F (Fay et al. 1997), Z1 (Soltis et al. 1992), rbcL2 forward (Palmieri et al. 2009), and R‐Parveen‐F (Parveen et al. 2012), which were used in 287 (44.36%), 144 (22.26%), 16 (2.4%), 11 (1.70%), and 8 (1.2%) of the papers, respectively (Figure 1A). The most frequently used reverse primers for the *rbcL* gene were rbcLaR (Kress et al. 2009), rbcL‐724R (Fay et al. 1997), 1326R (Cuénoud et al. 2002), rbcLajf634R (Fazekas et al. 2008), and aR (Kress and Erickson 2007) in 155 (23.95%), 146 (22.56%), 136 (21.02%), 60 (9.27%), and 24 (3.70%) of the papers, respectively (Figure 1B). Including forward and reverse primers, 294 primers were used in only one paper.

Primers for the *matK* gene were designed in 152 different papers. The primers developed by K. J. Kim (Unblished) and Cuénoud et al. (2002) were the most frequently used, both forward and reverse (Figure 1C,D). These forward primers 1RKIM and 390F were used in 192 (29.67%) and 145 (22.41%) papers, respectively (Figure 1C). The two most used reverse primers for the *matK* gene were 3FKIM (*N* = 198; 30.60%) and 1326R (*N* = 136; 21.02%) (Figure 1D). For both senses, 339 *matK* primers were used in only one paper.

### 3.2 Sequence Information

**FIGURE 2:**
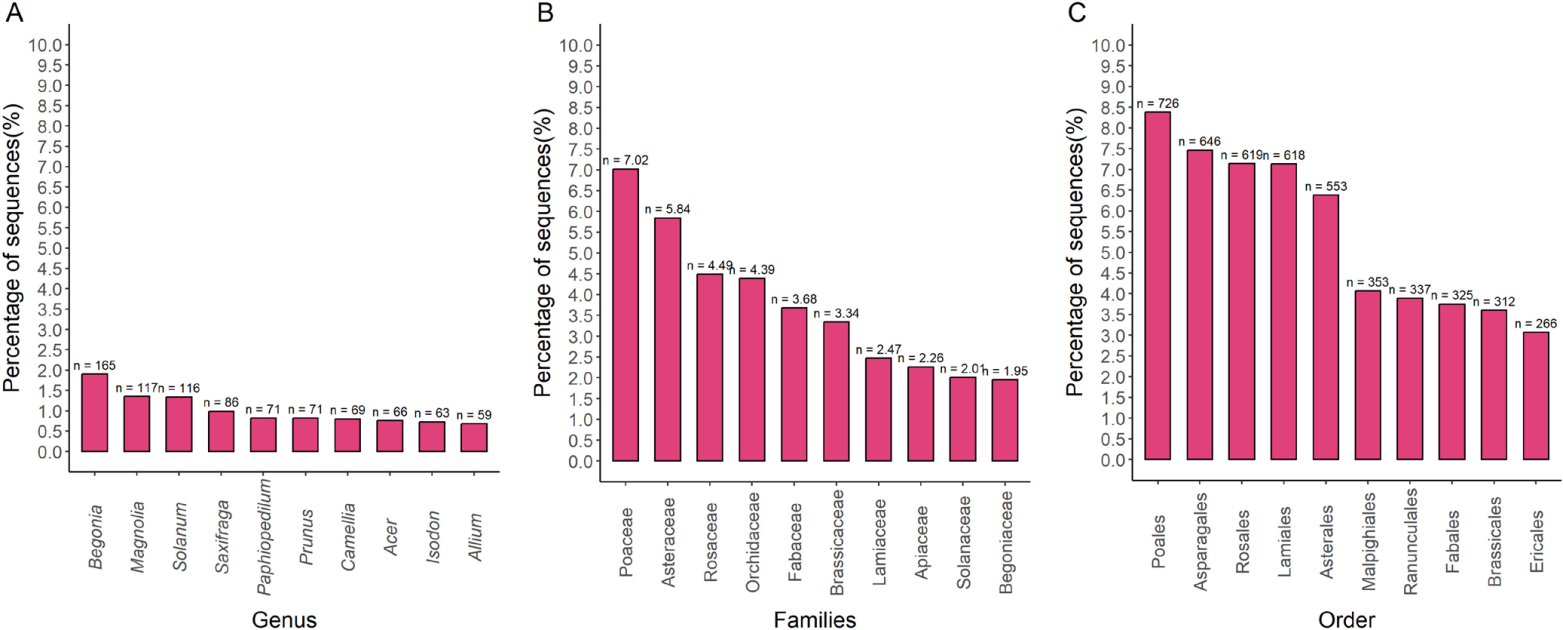
Top 10 (A) genus, (B) families, and (C) order with the most number of complete chloroplast genomes in the Refseq database (NCBI). The complete chloroplast genome genes *rcbL* and *matk* were used as template for *in silico* annealing evaluation.

The total number of 8859 land plants’ cpDNA sequences was available in June of 2022, of which only 8665 cpDNA passed through all filters. We recovered *rbcL* and *matK* gene sequences from 8463 different species, 2530 different genera, 342 different families, and 98 different orders (Table S3). The orders with the largest number of sequences were Poales (*N* = 726; 8.38%), Asparagales (*N* = 646; 7.4%), and Rosales (*N* = 619; 7.14%) (Figure 2C). The families Poaceae (*N* = 608; 7.02%), Asteraceae (*N* = 506; 5.84%), and Rosaceae (*N* = 389; 4.49%) have the highest number of sequences. The genera *Begonia*, *Magnolia*, and *Solanum* have the most sequences with 165 (1.90%), 117 (1.35%), and 116 (1.34%) sequences, respectively. Nineteen orders have only one sequence: Amborellales, Bartramiales, Buxbaumiales, Calobryales, Cardiopteridales, Ceratophyllales, Dicranales, Diphysciales, Funariales, Ginkgoales, Gleicheniales, Icacinales, Pallaviciniales, Pelliales, Psilotales, Ptilidiales, Salviniales, Tetraphidales, and Welwitschiales.

### 3.3 rbcL Coverage

**FIGURE 3:**
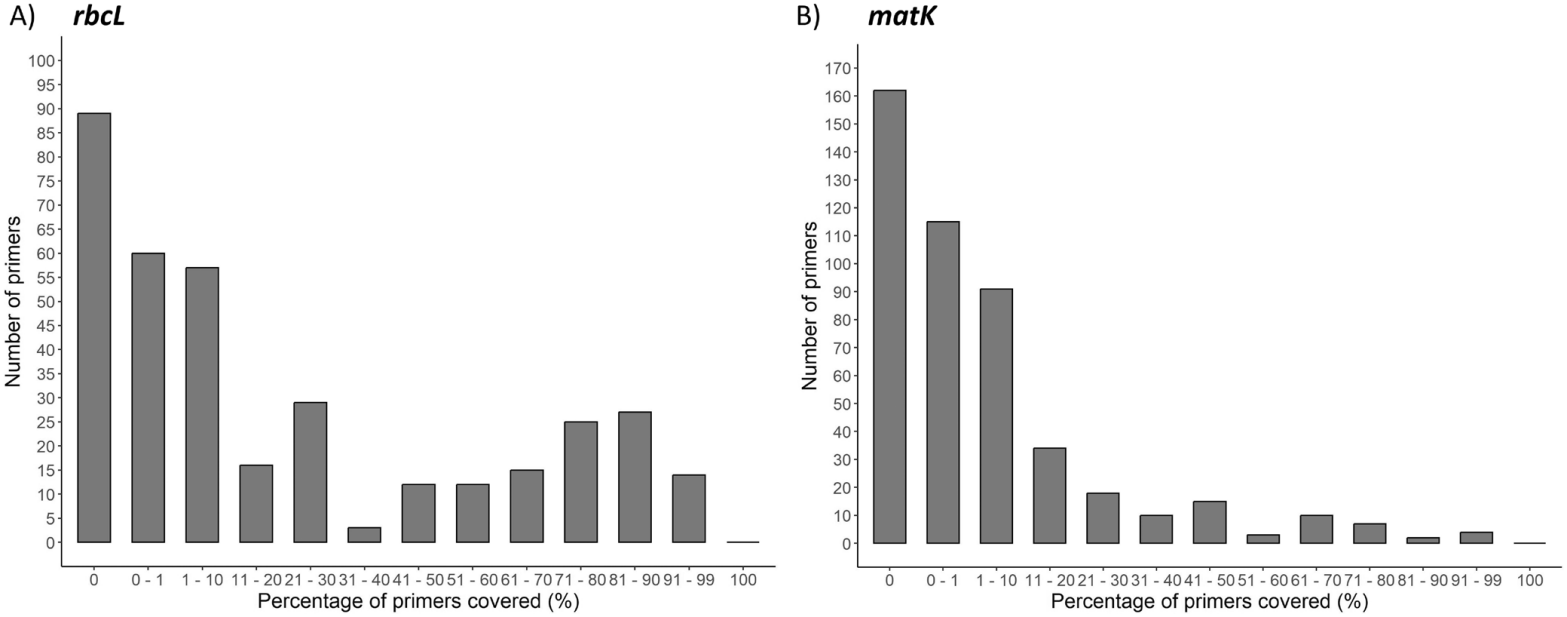
Number of *rbcL* and matK primers that covered the intervals of percent covered sequences. (A) *rbcL* primers and (B) *matK primers*.

No primer (forward and reverse) was able to amplify all sequences or at least one sequence from each order (Figure 3A) in the *in silico* evaluation. Considering the maximum number of mismatches of 7, the majority of primers did not cover any sequence or covered < 1% of the sequences (Figure 3A). Of the 366 *rbcL* primers, 89 did not cover any sequence, and 60 primers covered < 1% of the sequences. On the other hand, 27 primers covered between 81% and 90% of the sequences, and 14 primers covered between 91% and 99% of the sequences (Figure 3A). Evaluating the forward primers, the primers H1f (Fofana et al. 1997) and rbcL_4_For (Christin et al. 2011) can amplify the greatest number of sequences, both amplifying 8352 sequences (96.39%) and 87 covering orders at least one sequence per order (Table 1). Within the reverse primers, the primer with the greatest number of sequences covered was rbcLF reverse (Palmieri et al. 2009), which covered 8129 sequences (93.81%) and covered at least one sequence in 95 orders (Table 1).

**TABLE 1:** Top five *rbcL* primers (forward and reverse) with the largest number of sequences covered.

Forward						Reverse					
Primer	Sequence	*N*	*N%*	NO	NO%	Primer	Sequence	*N*	*N%*	NO	NO%
H1f (Fofana et al. 1997)	CCACAAACAGAGACTAAAGC	8352	96.39	87	88.77	rbcLF reverse (Palmieri et al. 2009)	ATATGCCAAACRTGRATACC	8129	93.81	95	96.93
rbcL_4_For (Christin et al. 2011)	TCACCACAAACAGARACTAAAGC	8352	96.39	87	88.77	rbcLE reverse (Palmieri et al. 2009)	TGATCTCCACCAGACAKACG	7643	88.21	74	75.51
R‐Parveen‐F (Parveen et al. 2012)	ATGTCACCACAAACAGAAACTAAAGC	8351	96.38	87	88.77	rbcL‐3‐R (Jiao et al. 2015)	TTCCCCTTCAAGTTTACC	7384	85.22	90	91.83
rbcL F (Kress and Erickson 2007)	ATGTCACCACAAACAGAGACTAAAGC	8350	96.36	87	88.77	rbcLA (Palmieri et al. 2009)	CCTTTRTAACGATCAAGRC	6948	80.18	82	83.67
ESRBCL1FAS (Schneider and Schuettpelz 2006)	ATGTCACCACAAACGGAGACTAAAGC	8349	96.35	87	88.77	rbcL 840R (Yamashita and Tamura 2000)	TTGTCGCGGCAATAATGAGCC	6685	77.15	70	71.42

Abbreviations: N, number of sequences covered; N%, percentage of covered sequences; NO, number of orders with at least one covered sequence.

Although we detected a positive correlation between the number of sequences covered across primer number of orders covered, for both forward (*r* = 0.9260; *p* < 0.01) and reverse (*r* = 0.8695; *p* < 0.01), we identified different ranks in primers sorted by number of sequences and orders covered. The ranking of the top five primers forward and reverse changed when we used the ranking criteria of number of orders covered (Table 2). For instance, none of the 5 forward primers with the highest number of orders covered are included in the top 5 forward primers ranked by the number of sequences covered (Tables 1 and 2). We have highlighted that the rbcLF reverse (Palmieri et al. 2009) primer has a larger number of sequences and orders covered. These changes may be related to the variation in the number of sequences per order and the preferred group for amplification. At least one sequence for each primer is provided in Table S4, indicating which primer can be used for each order.

**TABLE 2:** Top *rbcL* five primers (forward and reverse) with the largest number of orders covered.

Forward						Reverse					
Primer	Sequence	*N*	*N%*	NO	NO%	Primer	Sequence	*N*	*N%*	NO	NO%
rbcL‐640‐F (Gradstein et al. 2006)	CTCAACCATTTATGCGTTGG	7698	88.84	93	94.89	rbcLF reverse (Palmieri et al. 2009)	ATATGCCAAACRTGRATACC	8129	93.81	95	96.93
Ce 622 (Daugbjerg et al. 1994)	TCACAACCATTTATGCGTTG	7699	88.85	93	94.89	rbcLbR (Dong et al. 2014)	TCGGTYAGAGCRGGCATRTGCCA	4869	56.19	93	94.89
rbcL‐127F (Su et al. 2008)	CTGCGGTAGCTGCCGAATCTTC	8153	94.09	91	92.85	rbcL‐556R (Aziz et al. 2017)	ACATTCATAAACHGCYCTACC	5830	67.28	91	92.85
rbcLbF (Dong et al. 2014)	AGACCTWTTTGAAGAAGGTTCWGT	7323	84.51	90	91.83	rbcL‐3‐R (Jiao et al. 2015)	TTCCCCTTCAAGTTTACC	7384	85.22	90	91.83
HRM_rbcL3F (Srivastava et al. 2018)	TAGACCTTTTTGAAGAAGGTTCTGT	7300	84.25	90	91.83	r‐Guo‐r (Guo et al. 2019)	TCGGTYAGAGCRGGCATATGCCA	4731	54.60	88	89.79

Abbreviations: N, number of sequences covered; N%, percent of sequences covered; NO, number of orders with at least one amplified sequence.

The *rbcL* sequence of *Galeola lindleyana* (NC_064997.1, Asparagales) was the only sequence not covered by two or more primers. The other 8462 sequences have been covered by > 14 primers. The most covered species were *Actinodaphne cupularis* (NC_062885.1, Laurales), *Dehaasia hainanensis* (NC_068504.1, Laurales), *Dodecadenia grandiflora* (NC_070175.1, Laurales), *Sassafras randaiense* (NC_072676.1, Laurales), *Phoebe yaiensis* (NC_079582.1, Laurales), *Phoebe hungmoensis* (NC_079583.1, Laurales), and *Hesperomeles goudotiana* (NC_045327.1, Rosales), all covered by 117 primers.

**FIGURE 4:**
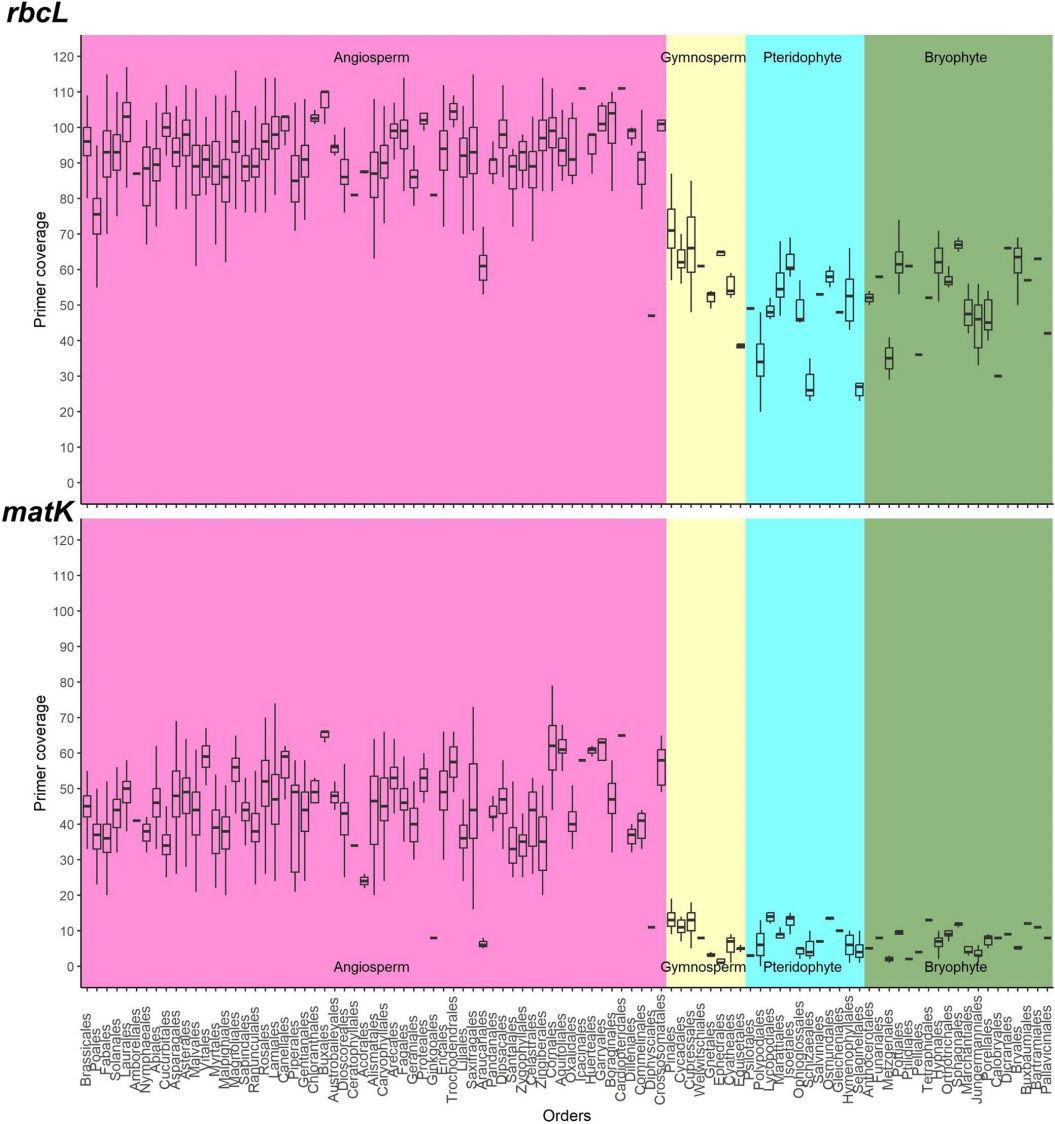
Boxplot showing the number of *rbcL* and *matK* primers covered each sequence per order.

No order was not covered by at least one primer pair. The average primer coverage was 89.71%, and the order with the highest average primer coverage was Cardiopteridales (*n* = 1) and Icacinales (*n* = 1) both with 111 primers, and the lowest primer coverage was 28.14 primers for the order Selaginellales (*n* = 7) (Figure 4; Table S5). When analyzing the Pearson correlation between the number of sequences per order and the number of primers covered, we identified a lower significant correlation (*r* = 0.3482; *p*‐value < 0.01).

**FIGURE 5:**
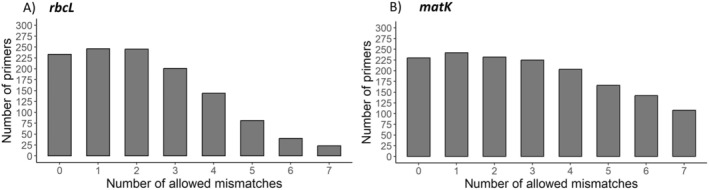
Number of *rbcL* and *matK* primers that are bound to at least one sequence with different numbers of allowed mismatches. (A) *rbcL* primers and (B) *matK primers*.

Using the relationship between the primers and the templates, it was possible to identify the number of mismatches and the number of unaligned nucleotides between the primer and template sequence. Only 233 primers showed no mismatches when evaluating exclusively the primer‐sequence relationships. Results in no mismatches, 233 primers (forward and reverse) (Figure 5A). We observed that when the number of allowed mismatches was one, the highest number of primers was recovered (246 primers with at least one binding event) (Figure 5A). The number of sequences amplified for each primer changes when compared to the number of mismatches allowed (Table S2). When the number of allowed mismatches is zero, the reverse primer rbcLF reverse (Palmieri et al. 2009) and the forward primer 1 forward (Vidal‐Russell and Nickrent 2019) cover the most sequences, with 7903 (94.24%) and 7768 sequences covered (93.01%), respectively (Table S6).

We also evaluated the PCR physicochemical properties for each primer. The forward and reverse primers ranged in length from 14 to 33 bp. (Table 3). The reverse primers have a higher average melting temperature of 54.55°C (SD = 4.93) and a GC ratio of 0.46 (SD = 0.08) than the forward primers with an average melting temperature of 52.38°C (SD = 4.79) and a GC ratio of 0.43 (SD = 0.08). For both forward and reverse primers, the number of GCs at the 3′ end (GC clamp) ranged from 0 to 5 (Table 3). The average number of homopolymer runs (N runs) in forward primers was 3.04 (SD = 0.76) and in reverse primers was 3.03 (SD = 0.83) (Table 3). No repeats were detected in reverse primers. Most forward and reverse primers showed forming energy in a secondary structure equal to 0.

**TABLE 3:** PCR physicochemical properties’ and primer properties’ descriptive analysis for *rbcL* primers.

	Tm (°C)	Length	Degeneracy	GC ratio	GC clamp	N runs	N repeats	∆G
Forward primers								
Average	52.28	22.32	1.28	0.43	0.82	3.04	1.77	−0.49
Median	52.49	22.00	1.00	0.42	1.00	3.00	2.00	0.00
SD	4.79	3.09	1.00	0.08	0.97	0.76	0.65	0.96
Maximum	68.66	34.00	12.00	0.71	5.00	5.00	4.00	0.00
Minimum	36.53	14.00	1.00	0.25	0.00	2.00	1.00	−5.82
Reverse primers								
Average	54.55	22.46	1.64	0.46	1.14	3.03	0.00	−0.37
Median	54.50	22.00	1.00	0.46	1.00	3.00	0.00	0.00
SD	4.93	3.19	1.69	0.08	1.26	0.83	0.00	0.69
Maximum	71.52	33.00	12.00	0.67	5.00	5.00	0.00	0.00
Minimum	35.73	15.00	1.00	0.16	0.00	2.00	0.00	−3.27

Abbreviations: Degeneracy, number of degenerative bases; ∆G, energy of secondary structure formation shown in kcal/mol; GC ratio, percentage of guanine and cytosine in the primer sequence; GC clamp, number of guanine and cytosine in the primer 3′ end; N runs, number of homopolymers; N repeats, number of dinucleotide repeats; SD, standard deviation; Tm, melting temperature.

A total of 76 primers survived the eight constraints filter, of which 33 were forward primers and 43 were reverse primers. Therefore, we identified the following forward primers as having the largest in the number of sequences bound to them: rbcL_4_For (Christin et al. 2011) (*n* = 8352; 96.39%), rbcL F (Kress and Erickson 2007) (*n* = 8350; 96.36%), and rbcL‐26‐F (Iida et al. 2007) (*n* = 8348; 96.34%), while the reverse primers were rbcLF reverse (Palmieri et al. 2009) (*n* = 8129; 93.81%), rbcLE reverse (Palmieri et al. 2009) (*n* = 7643; 88.21%), and rbcL‐556R (Aziz et al. 2017) (*n* = 5830; 67.28%) (Table 4). The GC clamp and primer coverage filters were responsible for filtering out the largest number of primers, where 170 (47.35%) primers did not have between 0 and 4 guanine or cytosine in the 3′ end, and 156 (43.43%) primers did not cover one or more sequences.

**TABLE 4:** Top ten *rbcL* primers with the largest number of sequences covered and that passed in all filtering constraints.

Forward						Reverse					
Primer	Sequence	*N*	*N%*	NO	NO%	Primer	Sequence	*N*	*N%*	NO	NO%
rbcL_4_For (Christin et al. 2011)	TCACCACAAACAGARACTAAAGC	8352	96.39	87	88.78	rbcLF reverse (Palmieri et al. 2009)	ATATGCCAAACRTGRATACC	8129	93.81	95	96.94
rbcL F (Kress and Erickson 2007)	ATGTCACCACAAACAGAGACTAAAGC	8350	96.36	87	88.78	rbcLE reverse (Palmieri et al. 2009)	TGATCTCCACCAGACAKACG	7643	88.21	74	75.51
rbcL‐26‐F (Iida et al. 2007)	TGTCACCACAAACAGAGACTAAAGC	8348	96.34	87	88.78	rbcL‐556R (Aziz et al. 2017)	ACATTCATAAACHGCYCTACC	5830	67.28	91	92.86
Z1aF (Hofreiter et al. 2000)	ATGTCACCACCAACAGAGACTAAAGC	8343	96.28	87	88.78	HRM_rbcL1R (Srivastava et al. 2018)	TCCACACAGTTGTCCATGTACC	4521	52.18	80	81.63
1 forward (Vidal‐Russell and Nickrent 2019)	ATGTCACCACAAACAGARAC	8339	96.24	68	69.39	h1aR (Poinar et al. 1998)	GAGGAGTTACTCGGAATGCTGCC	4146	47.85	61	62.24
Pteridaceae_rbcLF (Park et al. 2020)	CCACAAACGGAGACTAAAGC	8336	96.20	87	88.78	rbcLD reverse (Palmieri et al. 2009)	TAGTATTTGCDGTGAATCCC	3155	36.41	47	47.96
217F (Pirie et al. 2005)	GGACTTACCAGCCTTGATCG	8255	95.27	88	89.80	r‐Abbasi‐R (Abbasi et al. 2019)	GCTTCGGCACAAAAKARGAARCGGTCTC	2404	27.74	56	57.14
r‐CBOL‐? (CBOL Plant Working Group 2009)	ATGTCACCACAAACAGARACTAAAGC	8108	93.57	87	88.78	RrbcLb‐cr (Xu et al. 2015)	ATGCCCTTTGATTTCACCTGTTTC	2378	27.44	66	67.35
ke‐F (Kress and Erickson 2007)	CTTACCAGYCTTGATCGTTACAAAGG	7478	86.30	76	77.55	rbcL‐5‐R (Jiao et al. 2015)	CACGAGCAAGATCACGTC	2321	26.79	64	65.31
rbcL1 (Palmieri et al. 2009)	GCAGCATTCCGAGTAACTCCTCA	7445	85.92	59	60.20	rbcL‐724R (Fay et al. 1997)	TCGCATGTACCTGCAGTAGC	2014	23.24	62	63.27

Abbreviations: N, number of sequences covered; N%, percentage of sequences covered; NO, number of orders with at least one covered sequence; NO%, percentage of orders covered.

### 3.4 matK Coverage

We do not show any primer (forward and reverse) with *in silico* ability to amplify > 79% of the orders. Among the 490 available primers, only 4 primers covered > 90% of the sequences, all of which were forward primers (Figure 3B). Most primers (*N* = 162) did not cover any sequences, and 115 primers covered < 1% of the sequences (Figure 3B). The forward primers showed higher‐capacity *in silico* amplification. For example, the best forward primer (ability to amplify a greater number of sequences), matK‐XF (Dunning and Savolainen 2010), bound to 91.56% of the sequences (Table 5), while the reverse primers 3FKIM (K. J. Kim, unpublished data), 1326R‐2 (Cuénoud et al. 2002), and matK‐1227r‐6 (Heckenhauer et al. 2016) bound to 61.62%, 58.77%, and 36.65% of the sequences (Table 5).

**TABLE 5:** Top five *matK* primers (forward and reverse) with the largest number of sequences covered.

Forward						Reverse					
Primer	Sequence	*N*	*N%*	NO	NO%	Primer	Sequence	*N*	*N%*	NO	NO%
matK‐XF (Dunning and Savolainen 2010)	TAATTTACGATCAATTCATTC	7934	91.56	75	76.53	3FKIM (K. J. Kim, unpublished data)	CGTACAGTACTTTTGTGTTTACGAG	5339	61.62	53	54.08
matK‐F_Poalesv2 (Loera‐Sánchez et al. 2020)	GAATTTACGCTCTATTCAKTC	7899	91.16	74	75.51	matK‐1227r‐6 (Heckenhauer et al. 2016)	GARGATCCGCTRTAATAATGCGAAAGATTT	3176	36.65	51	52.04
XF4 (Dunning and Savolainen 2010)	TAATTTACGATCAATTCATKC	7878	90.92	78	79.59	matK‐XF 2 (Dunning and Savolainen 2010)	ACAAGAAAGTCGAAGTAT	2503	28.89	60	61.22
XF1 (Dunning and Savolainen 2010)	TAATTTACGATCAATTCAKTC	7872	90.85	76	77.51	LAM_R (Dunning and Savolainen 2010)	GCACAAGAAAGTCGAAGTATATA	2375	27.41	69	70.40
Adiantum_For (Ghahramanzadeh et al. 2013)	GATGTTGCAGTCTATTCATTC	7614	87.87	72	73.46	1300R (Samuel et al. 2005)	CGAAGTATATAYTTYATTCGATACA	2281	26.32	50	51.02

Abbreviations: N, number of sequences covered; N%, percentage of sequences covered; NO, number of orders with at least one covered sequence; NO%, percentage of orders covered.

When comparing the primers with the higher amplification capability, based on the number of orders and number of sequences, our results show different top five primers. The primers with the most number of orders, with at least one covered sequence, are XF4 (Dunning and Savolainen 2010) and XF1 (Dunning and Savolainen 2010) for forward primers, binding to 78 and 76 orders, respectively. For reverse primers, the top rank was LAM_R (Dunning and Savolainen 2010) and matK‐XF 2 (Dunning and Savolainen 2010), binding to 69 and 60 orders, respectively. These results opposed the expected by the Person correlation between the number of sequences covered and the number of orders covered, which determines a positive and significant correlation for forward primers (*r* = 0.9158; *p*‐value < 0.01) and reverse primers (*r* = 0.8325; *p*‐value < 0.01). Here, we also indicated the orders by each primer for covering an order the primer should cover at least one sequence (Table S7).

Only two sequences were not covered by any primer, the *matK* genes of *Bolbitis sinensis* (NC_071924.1, Polypodiales) and *Bolbitis tonkinensis* (NC_071958.1, Polypodiales). In total, 16 species are covered by only one primer. For the orders, each order has at least one primer covering at least one sequence, that is, a species with a *matK* gene covered by one primer. The orders Cardiopteridales (*n* = 3) and Buxales (*n* = 1) have the highest average primer coverage with 65 primers, and the order Ephedrales (*n* = 5) has the lowest average primer coverage with 1.4 primers (Figure 4; Table S8). The standard variation in the primer coverage per order ranges from 12.87 (Piperales, *n* = 27) to 0.55 (Ephedrales, *n* = 5). Among the orders with more than five sequences, our results also indicate the presence of outlier species (Figure 4; Table S8). We tested for a possible correlation between the number of sequences and the number of primers covered, which was positive and significant (*r* = 0.37; *p*‐value < 0.01).

Comparing the primers and their templates, we counted the number of allowed mismatches. The *matK*, just as the *rbcL*, showed the biggest number of primers bound to templates with one mismatch allowed (*n* = 243), followed by 0 and 2 mismatches, both with 233 and 231 primers bound to at least one template sequence, respectively (Figure 5B). For these numbers of allowed mismatches of *matK*, we found a similar profile to that of *rbcL*, with a decrease in the number of bound primers as the number of mismatches increases. Allowing no mismatches, the primers matK‐XF (Dunning and Savolainen 2010) and 3FKIM (K. J. Kim, unpublished data) covered the most number of sequences covering 2866 sequences (33.86%) and 2111 (24.93%) sequences, respectively (Table S9).

We evaluated the properties of each forward and reverse *matK* primer, and they showed a median length of 22 bp, ranging from 35 to 15 bp for forward primers and 30 to 13 bp for reverse primers (Table 6). The *matK* primers, in general, showed lower melting temperature than *rbcL* primers, with an average melting temperature of 49.73°C (SD = 5.03) for forward primers and 50.60°C (SD = 4.98) for reverse primers (Table 6). The GC ratio and GC clamp for forward primers ranged from 0.65 to 0.18 and 5 to 0, respectively. For the GC ratio and clamp in reverse primers, the values ranged from 0.61 to 0.18 and 4 to 0, respectively (Table 6). The energy for forming secondary structure (∆G) for forward and reverse primers ranged from −6.61 to 0, with an average −0.49 (SD = 0.96) for forward primers and of −0.40 (SD = 0.70) for reverse primers.

47 *matK* primers remained after the constraint filter, of which 23 were forward and 24 were reverse primers. Our results show that not all primers with higher sequence and order coverage (Tables 5 and 7) have the desired metrics to fulfill all physicochemical constraints. Therefore, the forward primers that passed the physicochemical evaluation and covered the largest number of template sequences were matK472F (Yu et al. 2011) (45.79%; *n* = 3968), m‐Yu‐F (Yu et al. 2011) (44.89%; *N* = 3890), and 300F (Harris et al. 2009) (27.37%; *N* = 2372) while for reverse primers were 3FKIM (K. J. Kim, unpublished data) (61.62%; *N* = 5339), 1326R‐2 (Cuénoud et al. 2002) (58.77%, *N* = 5092), and 1440R (Fior et al. 2006) (19.67%; *N* = 1704) (Table 8).

**TABLE 6:** PCR physicochemical properties’ and primer properties’ descriptive analysis for *matK* primers.

	Tm (°C)	Length	Degeneracy	GC ratio	GC clamp	N runs	N repeats	∆G
Forward primers								
Average	49.73	22.09	1.45	0.39	0.87	3.12	1.65	−0.49
Median	52.49	22	1	0.42	1	3	2	0
SD	5.03	2.67	1	0.09	0.90	0.73	0.5	0.96
Maximum	62.21	35	12	0.65	5	5	3	0
Minimum	35.65	15	1	0.18	0	1	1	−6.61
Reverse primers								
Average	50.60	21.84	1.416309	0.41	0.82	3.10	0	−0.40
Median	54.5	22	1	0.45	1	3	0	0
SD	4.98	3.0	1.25	0.08	0.94	0.76	0	0.70
Maximum	61.25	30	8	0.61	4	5	0	0
Minimum	33.93	13	1	0.19	0	2	0	−3.91

Abbreviations: Degeneracy, number of degenerative bases; ∆G, energy of secondary structure formation shown in kcal/mol; GC ratio, percentage of guanine and cytosine in the primer sequence; GC clamp, number of guanine and cytosine in the primer 3′ end; N runs, number of homopolymers; N repeats, number of dinucleotide repeats; SD, standard deviation; Tm, melting temperature.

**TABLE 7:** Top five *matK* primers (forward and reverse) with the largest number of orders covered.

Forward						Reverse					
Primer	Sequence	*N*	*N%*	NO	NO%	Primer	Sequence	*N*	*N%*	NO	NO%
XF4 (Dunning and Savolainen 2010)	TAATTTACGATCAATTCATKC	7878	90.92%	78	79.59	LAM_R (Dunning and Savolainen 2010)	GCACAAGAAAGTCGAAGTATATA	2375	27.41	69	70.40
XF1 (Dunning and Savolainen 2010)	TAATTTACGATCAATTCAKTC	7872	90.85%	76	77.55	matK‐XF 2 (Dunning and Savolainen 2010)	ACAAGAAAGTCGAAGTAT	2503	28.89	60	61.22
matK‐XF (Dunning and Savolainen 2010)	TAATTTACGATCAATTCATTC	7934	91.56%	75	76.53	ERI_R (Dunning and Savolainen 2010)	GCACAAGAAAGTCGAAGTAT	2100	24.24	59	60.20
matK‐F_Poalesv (Loera‐Sánchez et al. 2020)	GAATTTACGCTCTATTCAKTC	7899	91.16%	74	77.51	matK‐MALP‐R1 (Dunning and Savolainen 2010)	ACAAGAAAGTCGAAGTA	2004	23.13	58	59.18
Adiantum_For (Ghahramanzadeh et al. 2013)	GATGTTGCAGTCTATTCATTC	7614	87.87%	72	73.47	AST_R (Dunning and Savolainen 2010)	CAAATAATATCCAAATACCAA	2094	24.17	55	56.12

Abbreviations: N, number of sequences covered; N%, percentage of sequences covered; NO, number of orders with at least one covered sequence.

**TABLE 8:** Top 10 *matK* primers with largest number of sequences covered and that passed in all filtering constraints.

Forward						Reverse					
Primer	Sequence	*N*	*N%*	NO	NO%	Primer	Sequence	*N*	*N%*	NO	NO%
matK472F (Yu et al. 2011)	CCCRTYCATCTGGAAATCTTGGTTC	3968	45.79	50	51.02%	3FKIM (K. J. Kim, unpublished data)	CGTACAGTACTTTTGTGTTTACGAG	5339	61.62	53	54.08%
m‐Yu‐F (Yu et al. 2011)	GTCCATGTCGAAATCTTGGTTC	3890	44.89	49	50.00%	1440R (Fior et al. 2006)	GTGTTTACGAGCYAAAGTTC	1704	19.67	31	31.63
300F (Harris et al. 2009)	GGGATTTGCAGTCATTGTGG	2372	27.37	40	40.82%	FAB_R (Dunning and Savolainen 2010)	CTTTTGTGTTTACGAGCCAADG	1431	16.51	48	48.98
matK‐Aris‐F458 (Dechbumroong et al. 2018)	ATACCCCACCCCATCCATCTG	1712	19.76	40	40.82%	8R2 (Wang et al. 2007)	ACGWGCCAAAGTTCTAGCAC	1177	13.58	30	30.61
matK743F (Whitten et al. 2000)	CTTCTGGAGTCTTTCTTGAGC	1694	19.55	35	35.71%	M‐Parveen‐R (Parveen et al. 2012)	GTTCTAGCACACGAAAGTCG	541	6.24	30	30.61
matk‐2051f‐br (Barfuss et al. 2016)	GTATCGGGACATCCTATTAGTAAGCC	1654	19.09	41	41.84%	4R (Bremer et al. 2002)	GCATCTTTTACCCARTAGCGAAG	400	4.62	19	19.39
F389 (de Vere et al. 2012)	GGAAATCCATTCTGGCTTCAAAAGG	1526	17.61	23	23.47%	R2caryophyllales (de Vere et al. 2012)	TGTGTTTACGAGCCAAAGTTCTAGC	272	3.14	21	21.43
matK‐13F (Su et al. 2008)	CTAATACCTCACCCCGTCCATCTG	1295	14.95	40	40.82%	matKKUr (Srikulnath et al. 2015)	CGAGCCAAAGTTCTAGCACACG	245	2.83	12	12.24
MF (Xu et al. 2013)	TCCTACCGTGTGTGAATGCG	740	8.54	27	27.55%	500R (Harris et al. 2009)	TGGACRGGRTRGGGTATTAG	130	1.50	23	23.47
matk‐1610f‐br (Castello et al. 2016)	AACATCTTCTGGAACCTTTCTTGAGCG	690	7.96	29	29.59%	R1460poales (de Vere et al. 2012)	AGGGTTGTTTTGGTGAACATCAAAG	128	147	1	1.02

Abbreviations: N, number of sequences covered; N%, percentage of sequences covered; NO, number of orders with at least one covered sequence.

### 3.5 Optimal Set of Primers

**FIGURE 6:**
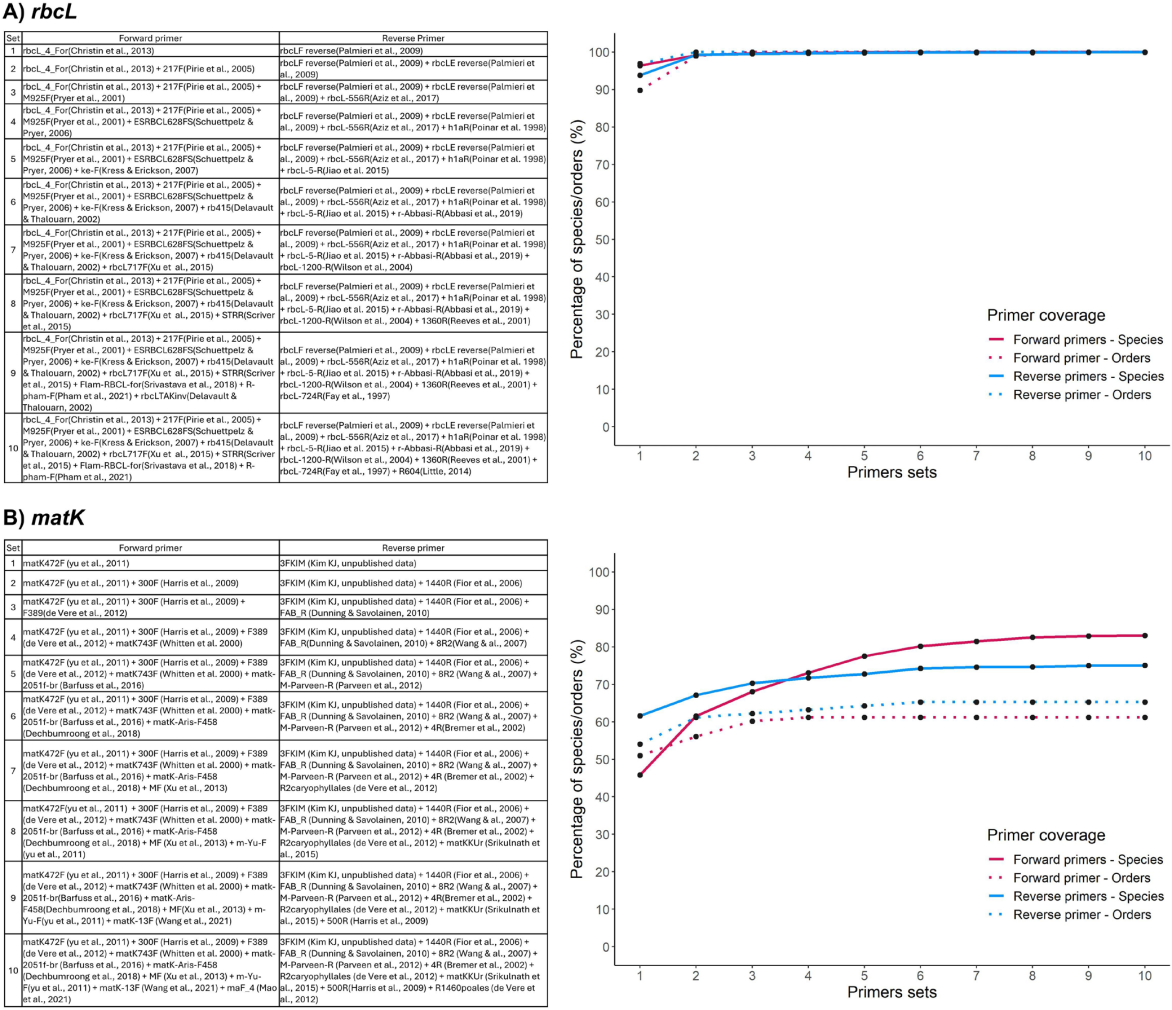
Optimal primer sets for greater sequence and order coverage for both *rbcL* and *matK* genes.

Our result shows that the *rbcL* forward primers can bind to 99.96% of the sequences (8662) and the reverse primers to 99.89% of the sequences (8656) when using a set of 6 (forward) and 10 (reverse) primers. However, using only a set of 2 two primer pairs, the result is > 99% of sequence coverage (Figure 6A). For the order number with at least one amplified sequence, our simulation indicated all orders will be amplified with 2 two primer forwards and 3 primer reverses (Figure 6A).

For the *matK* gene, with the largest optimal primer set tested (10 primers), we obtained a total of 83.01% of the sequences covered by forward primers (7193) and 75.08% of the sequences covered by reverse primers (6506). Evaluating the number of orders with at least one covered sequence with ten primers, 61.22% were covered by forward primers and 65.30% were covered by reverse primers (Figure 6B). The number of orders covered by forward primers ranged from 51.02% (one primer) to 61.22% (four primers or more). The set 1 of reverse primers covered 54.08% (53) of the orders, with a maximum coverage percentage obtained with six or more primers, corresponding to 65.30% (65) of the orders (Figure 6B).

## 4 Discussion

In this study, we revisited the primers commonly used in the literature for the amplification of two core plant DNA barcodes. Amount of 366 and 490 different primers for *rbcL* and *matK* were found and tested, respectively, in an *in silico* PCR with 8665 sequences (from 8463 species and 98 orders), to compare the primers’ ability to amplify the largest number of sequences and orders. We observed that the *rcbL* primers can amplify a larger number of sequences than the *matK* primers. Our result supports the idea of the existence of universal primers of *rbcL*. In opposition, the *matK* primers do not amplify a large number of species or orders, and we did not find a single *matK* universal primer. The result is alarming because, even with a set consisting of a combination of 10 *matK* primers, we were unable to recover information from a large part of the biodiversity of land plants.

We reviewed > 800 papers published before 2022, revealing a huge number of primers available, some of which were more frequently used than others, which is highly evidenced in *matK* region. Our results indicated that most primers, for both *matK* and *rbcL* regions, have an *in silico* amplification capability of < 10% of the sequences. Usually, these primers with low sequence coverage have been used for groups at lower phylogenetic scales (Park et al. 2020). In addition, the species‐specific primer has been successfully used to identify invasive species from environmental samples (Scriver et al. 2015). In our analyses, we used only sequences extracted from the complete chloroplast genome in the reference NCBI database (Refseq) to obtain the complete *rbcL* and *matK* genes. These complete gene sequences here were called template sequences. Since we only used full sequences, some species‐specific primers did not cover any sequences due to absence in the database.

A few sets of primers can amplify many orders or sequences. Although there is no consensus on how many amplified species for a taxon are necessary to consider a primer universal, this result may provide a first clue toward more comprehensive and universal evaluations. If we consider that for a primer to be universal for all the land plants, it is necessary to have a coverage of > 90% of the sequence templates, and only 14 primers fit these criteria and could be considered as “universal” for *rbcL*. For the *matK* region, this number of “universal” is reduced to 4 primers These universal primers have been successful when applied to species delimitation, species identification, or biodiversity monitoring using environmental samples through DNA barcode datasets (Bell et al. 2017; Dunning and Savolainen 2010; Girma et al. 2016; Liu et al. 2015).

Of the three pairs of *rbcL* primer with wider species coverage, only one was designed to be species‐specific (Fofana et al. 1997; Jiao et al. 2015; Kress and Erickson 2007; Palmieri et al. 2009). The primer rbcL*‐3‐R* was designed for the molecular identification of *Populus euphratica* wood samples (Jiao et al. 2015). The other five primers with wider species amplification, both forward and reverse, were designed to amplify taxonomic levels broader than the family (Fofana et al. 1997; Kress and Erickson 2007; Palmieri et al. 2009). It is worth highlighting that the R‐Parveen‐F primer is an adaptation of the primers described by Kress and Erickson (2007), since the referenced sequence for the R‐Parveen‐F primer is not the same as the referenced one (Knopf et al. 2012; Kress and Erickson 2007).

For the *matK* gene, among three pairs of primers (forward and reverse) with major sequence coverage, 4 primers (matK‐XF, matK‐1227r‐6, XF4, and matK‐XF 2) were designed for amplification of a large number of angiosperms (Dunning and Savolainen 2010; Heckenhauer et al. 2016). The reverse primer covering most of the sequences is unpublished (3FKIM). This primer is also the most widely used, utilized in a wide range of angiosperm taxonomic groups, even though the lack of a reference article prevents us from inferring which group this primer was designed for. The primers developed by Dunning and Savolainen (2010) to amplify the angiosperm orders represent 3 of the 6 most used primers. In addition, adaptations to the primers originally developed by Dunning and Savolainen (2010) have been made to increase their utility across the Poales order, resulting in the matK‐F_Poalesv2 primer being one of the primers with the highest number of amplified sequences (Loera‐Sánchez et al. 2020).

Our results indicate a correlation between the number of sequences covered and the number of orders with at least one covered template sequence. However, when considering the rank of primers with the highest number of sequences and number of orders, we detected changes in the rank of primers. This change may be associated with the divergent number of sequences in each order and the preferential application of the order. For example, the primer XF1 (Dunning and Savolainen 2010) is the second primer with the most number of orders covered but is the fourth primer in the number of template sequences covered. Modification in the XF1 (Dunning and Savolainen 2010) primer for major amplification in the Poales genera, the most represented order, made this primer now matK‐F_Poalesv2 (Loera‐Sánchez et al. 2020), one of the primers with the most sequence coverage capacity with a smaller number of orders covered (Loera‐Sánchez et al. 2020). This example shows a preferential potential coverage by the phylogenetic structure of the binding primer and the template sequences, a fundamental criterion for the choice of primers, especially in biodiversity access through DNA metabarcoding (Barnes and Turner 2016). In this case, the primers that potentially cover the largest number of template sequences may underestimate local diversity, since we have more species sequences but fewer orders recovered. The most recent biodiversity metrics applied to local conservation have a phylogenetic component (Winter et al. 2013), so in these cases, we recommend using the following primers: rbcL‐640‐F (Gradstein et al. 2006) and rbcLF reverse (Palmieri et al. 2009) for the *rbcL* region and XF4 (Dunning and Savolainen 2010) and LAM_R (Dunning and Savolainen 2010) for *matK*, which apply the largest number of potentially covered orders.

We tested a wide range of primers, resulting in a single template sequence from *Galeola lindleyana* that was not covered by any *rbcL* primer. *Galeola lindleyana* (family: Orchidaceae) is a mycoheterotrophic plant native to Asia (Zhou et al. 2023). The mycoheterotrophic is plants that do not need to realize photosynthesis (Wicke et al. 2014; Wicke and Naumann 2018; Zhou et al. 2023). Thus, the evolutionary process of their chloroplast genomes undergoes severe changes, with gene loss, pseudogenization, and total elimination of the mitochondrial genome (Wicke and Naumann 2018; Zhou et al. 2023). This process may involve modification of the *rbcL* gene sequence (Wicke and Naumann 2018; Zhou et al. 2023). Based on our criteria for selecting template sequences, we believe that many of these plants either lack the *rbcL* gene altogether or possess only pseudogenized remnants (< 1000 bp in size), necessitating a more cautious analysis when selecting a primer for parasitic and carnivorous plant species. For *matK*, only the species *Bolbitis sinensis* and *Bolbitis tonkinensis* did not match with any primer. *Bolbitis* (Dryopteridaceae) is a pantropical genus with terrestrial, lithophytic, or epiphytic plants and with few studies on the evolution of chloroplast genes (Wang et al. 2024). Factors that make it advisable to develop a primer specific to this genus.

Our analysis of the distribution of primer‐binding sites across orders reveals a stark contrast between the *rbcL* and *matK* regions. For *rbcL*, the order with the lowest average number of bound primers is 28.14 (Selaginellales), whereas for *matK* 42 orders have < 20 primers bound on average. Pinales, the order with fewer *matK* primers bound, is an important economic order with a significant number of sampled species, yet previous studies have encountered challenges in amplifying these regions (Saarela et al. 2013). However, this problem can be overcome by using more specific primers, such as those developed for gymnosperms, which have shown success in amplifying *matK* in this species (Saarela et al. 2013; Setsuko et al. 2023; Tan et al. 2018). Similarly, the order Polypodiales, characterized by a rich diversity of ferns, presents challenges for *matK* amplification despite its informational potential (Li et al. 2011). These findings underscore the need for tailored primer strategies, with universal *rbcL* primers excelling in amplification, while *matK* amplification in orders such as Pinales and Polypodiales requires more specific primer designs (Li et al. 2011; Saarela et al. 2013).

Looking at the number of primers covering each of the orders evaluated, we noticed a clear difference in the specificity of the primers for the *matK* and *rbcL* gene regions. While the orders have an average coverage of 28 *rbcL* primers, 42 orders have an average coverage of < 20 primers for the *matK* gene. Among these 42 orders, there are some orders of high economic importance, such as Pinales, for which primers are either being developed or have already been developed. Other orders, such as Polypodiales, which is well represented with > 100 sequences, have a small number of primers, which may require the development of new primers or the use of specific primers. It is worth mentioning that the genus *Bolditis* belongs to this group, which requires the development of more specific primers for this group.

When evaluating primer–template binding, it is important to consider that successful amplification involves more than just this interaction. Therefore, we evaluate physicochemical PCR properties, a robust method for assessing primer performance (Buck et al. 1999). Using eight constraints available for primer selection in OpenprimeR, we find that most primers fail to meet at least one of the criteria (Kreer et al. 2020). Notably, constraints related to the use of multiplex primers, such as cross‐dimerization energy, were not considered in the current analyses. Our results highlight GC clamp and coverage as key factors leading to primer exclusion. The GC clamp corresponds to the number of guanine and cytosine in the 3′ end of the primer and is one of the most important features for primer validation (Buck et al. 1999). This is important because the guanine and cytosine in the end 3′ guarantee high specificity due to the binding strength of guanine and cytosine (Apte and Daniel 2009). The coverage, the second criterion that more frequently excluded primers in our analysis, has a high number of primers not covering any template sequence. To evaluate primer coverage beyond their binding to the template sequence, three factors can restrict the primer coverage: (1) the free energy of annealing and the presence of 3′ end mismatches, (2) the thermodynamic model used, and (3) the introducion of stop codons (Kreer et al. 2020).

After filtering the sequences, it became clear that there was no single first pair of primers that amplified species across the entire biodiversity of terrestrial plants. We then tested a combination of primers that maximized the number of template sequences covered, making it advisable to use two primer pairs for *rbcL* broad taxonomic amplification: the forward primers rbcL_4_For (Christin et al. 2011) and 217F (Pirie et al. 2005) and the reverse primers rbcLF reverse (Palmieri et al. 2009) and rbcLE reverse (Palmieri et al. 2009), which altogether can recover > 99% of land plant sequences. This result highlighted this region as an important DNA barcode since previous studies showed a low ability to distinguish closely related species, and this region is important for deep phylogenetic reconstruction. On the other hand, for the *matK* gene, even when we use a primer set consisting of 10 pairs, we can cover < 85% of the template sequences, which is due to the high degree of variation in the *matK* gene region, resulting in a high degree of distinguishability between species (Burgess et al. 2011; Kress et al. 2009). This variation is also found in the annealing region of the primers, making it difficult to design truly universal primers for *matK* gene (Burgess et al. 2011; Kress et al. 2009). Given this difficulty, we recommend primers that cover most of the sampled orders and encourage studies that develop primers for the specific taxon in the *matK* region.

We provide valuable recommendations for the most appropriate primers to amplify a wide range of species. Our approach uses an *in silico* methodology, significantly reducing the time and cost of future studies. By employing *in silico* strategies, we can evaluate numerous primers against a wide range of sequences (de Melo et al. 2021; Kreer et al. 2020). In particular, our study represents the most comprehensive investigation to date, including a large number of primers and sequences tested for the *rbcL* and *matK* gene regions. Similar methods have previously been used to identify optimal primer sets for avians, focusing on the *COI* gene region (de Melo et al. 2021).

Our work aimed to evaluate the amplification success of the sequences, but the ability to distinguish species using these primers was not evaluated. However, previous studies have tested the species‐level discrimination ability of *matK* and *rbcL* data together, resulting in the successful identification of 72%–92% of angiosperm species in temperate and tropical regions (Burgess et al. 2011; CBOL Plant Working Group 2009; Kress et al. 2009; Setsuko et al. 2023). Although these two regions were chosen as core DNA barcode regions for various reasons, it is not expected that all plant diversity can be described by only two regions (CBOL Plant Working Group 2009). Therefore, other regions such as *ITs*, *trnH‐psbA*, and *atpF‐atpH* are increasingly being used and have not yet been studied *in silico* (Bell et al. 2017; Burgess et al. 2011; CBOL Plant Working Group 2009).

In this study, we conducted an extensive literature review to identify primers used to amplify two fundamental land plant DNA barcode regions. This resulted in a comprehensive *in silico* evaluation of primers targeting the *rbcL* and *matK* regions, comprising a total of 371 and 489 primers, respectively, along with 8859 template sequences representing 8463 different species. Our results show that no single primer has universal applicability across all terrestrial plant taxa. Nevertheless, among the evaluated primers, rbcL_4_For (Christin et al. 2011) and rbcLF reverse (Palmieri et al. 2009) emerge as the most promising candidates for universal amplification, particularly for terrestrial plant biodiversity studies. We observed notable differences in amplification efficiency between the *rbcL* and *matK* genes, with *rbcL* primers exhibiting broader taxonomic coverage compared to *matK* primers, which show greater specificity at the order, family, or genus level. Although *matK* shows promise as a DNA barcode region with high species identification potential, the challenges associated with amplification require careful selection of primers tailored to specific taxonomic groups or the development of novel primer sets.

## Author Contributions

**Leonardo C. J. Corvalán:** data curation (lead), formal analysis (lead), methodology (equal), software (lead), validation (lead), visualization (lead), writing – original draft (lead), writing – review and editing (equal). **Amanda A. de Melo‐Ximenes:** investigation (equal), methodology (equal), software (equal), validation (equal), writing – review and editing (equal). **Larissa R. Carvalho:** data curation (equal), formal analysis (equal), writing – review and editing (equal). **Carlos de M. e Silva‐Neto:** investigation (equal), writing – review and editing (equal). **José A. F. Diniz‐Filho:** conceptualization (equal), investigation (equal), writing – review and editing (equal). **Mariana P. de C. Telles:** conceptualization (equal), methodology (equal), writing – review and editing (equal). **Rhewter Nunes:** conceptualization (lead), data curation (equal), methodology (lead), project administration (equal), supervision (lead), writing – original draft (equal), writing – review and editing (equal).

## Conflicts of Interest

The authors declare no conflicts of interest.

## Supporting information


**Table S1.** Land plant barcode studies are available in the Web of Science database.

**Table S2.** Primer used in each DNA barcode study article.

**Table S3.** *rbcL* and *matK* sequence extracted from chloroplast genomes available in the reference database from NCBI and the corresponding taxonomic information of each sequence.

**Table S4.** Orders with at least one *rbcL* primer bound to its sequence.

**Table S5.** Descriptive metric of the primer number binds each sequence by order in the *rbcL* gene region.

**Table S6.** Number of mismatches for each *rbcL* primer sequence binded.

**Table S7.** Orders with at least one *matK* primer bound to its sequence.

**Table S8.** Descriptive metric of the primer number binds each sequence by order in the *matK* gene region.

**Table S9.** Number of mismatches for each *matK* primer sequence bound.

## Data Availability

We studied published data from the National Center for Biotechnology Information (NCBI) and published a paper from Web of Science (WoS). The NCBI ID reference and papers’ DOI are attached on Supporting Information Table.
